# Nasopharyngeal Wash with Normal Saline Decreases SARS-CoV-2 Viral Load: A Randomized Pilot Controlled Trial

**DOI:** 10.1155/2022/8794127

**Published:** 2022-09-27

**Authors:** I. Pantazopoulos, A. Chalkias, G. Mavrovounis, I. Dimeas, S. Sinis, A. Miziou, E. Rouka, K. Poulas, K. Gourgoulianis

**Affiliations:** ^1^Department of Emergency Medicine, Faculty of Medicine, University of Thessaly, Larissa, Greece; ^2^Department of Respiratory Medicine, Faculty of Medicine, University of Thessaly, Larissa, Greece; ^3^Department of Anesthesiology, Faculty of Medicine, University of Thessaly, Larissa, Greece; ^4^Outcomes Research Consortium, Cleveland, OH 44195, USA; ^5^Faculty of Nursing, University of Thessaly, Larissa, Greece; ^6^Department of Pharmacy, University of Patras, Patras, Greece

## Abstract

**Background:**

Although great progress has been made over the past 2 years in the scientific understanding of the biology, epidemiology, and pathogenesis of severe acute respiratory syndrome coronavirus 2 (SARS-CoV-2), case morbidity and fatality rates remain a great concern and continue to challenge the healthcare resources worldwide as novel variants emerge. There is therefore an urgent need for affordable and readily available strategies to reduce viral transmission. Previous studies in non-COVID-19 patients have demonstrated that administration of low-salt (isotonic but 0.0375% Na) and isotonic saline (0.9% Na) solutions has been associated with an immediate, significant reduction in the microbial antigens and a related decline of microbial burden. The aim of the present study was to determine the effect of nasal washes with normal saline 0.9% on nasopharyngeal viral load and outcome in hospitalized patients with COVID-19 pneumonia.

**Methods:**

We performed a prospective, randomized, pilot, controlled trial in 50 patients with confirmed COVID-19 disease. Patients were randomized into two groups, the normal saline group (received normal saline 0.9% solution for nasopharyngeal wash) and the control group (no treatment). In the normal saline group, nasopharyngeal wash was performed every 4 hours for a 16-hour period. Twenty-four hours after the baseline nasopharyngeal swab (and 8 hours after the last wash in the normal saline group), a second nasopharyngeal swab was collected for the semiquantitative estimation of the SARS-CoV-2 viral load as determined by cycle threshold (Ct) values.

**Results:**

In the normal saline group, mean N gene Ct values increased significantly 24 hours after the baseline measurement [ΔCt_day2−day1_ = 1.87 ± 3.11 cycles, *p* = 0.007 (95% CI: 0.55 to 3.18)], indicating a decline in SARS-CoV-2 nasopharyngeal viral load by 8.9%. A significant decrease in mean N gene Ct values was observed in the control group, indicating an increase in viral load [ΔCt_day2-day1_ = −2.12 ± 2.66, *p* < 0.001 (95% CI: −3.20 to −1.05)] by 9.7%. The difference between the two groups 24 hours after admission and nasopharyngeal wash was 3.09 cycles (*p* = 0.005, 95% CI: 0.97 to 5.20).

**Conclusion:**

Nasal washes with normal saline effectively decreased the viral load during hospitalization and at follow-up.

## 1. Introduction

Severe acute respiratory syndrome coronavirus 2 (SARS-CoV-2) emerged in Wuhan, China, in December 2019, and became a pandemic within a few months. Although great progress has been made over the past 2 years in the scientific understanding of the biology, epidemiology, and pathogenesis of SARS-CoV-2, case morbidity and fatality rates remain a great concern and continue to challenge the healthcare resources worldwide as novel variants emerge [1]. There is therefore an urgent need for affordable and readily available strategies to reduce viral transmission.

The nose is the dominant initial site for SARS-CoV-2 infection and the reverse transcription-polymerase chain reaction assay of nasopharyngeal swab samples remains the gold standard testing method to detect the virus [2]. It has been demonstrated that SARS-CoV-2 transmitters have more than three times higher viral loads than nontransmitters in nasopharyngeal/throat samples [3] Furthermore, high SARS-CoV-2 viral load in nasopharyngeal swab samples is an independent predictor of mortality [4]. Therefore, any strategy or method that would reduce SARS-CoV-2 viral load could decrease transmission and mortality in exposed patients.

Nasopharyngeal washes could prevent the virus from inhabiting and replicating in the nasal and pharyngeal mucosa and has been suggested as a potential method to reduce symptoms, transmission, and viral shedding in various acute respiratory tract viral infections [5]. Ct values obtained from Real-time PCR (RT-PCR) testing on consecutive specimens collected from the same patient and performed using the same assay, in the same microbiology laboratory can provide a relative measure of viral quantity in the different respiratory specimens. The primary aim of the present study was to determine the effect of nasal washes with normal saline 0.9% on nasopharyngeal viral load in hospitalized patients with COVID-19 pneumonia. The secondary aim was to examine if this effect influences escalation to high flow nasal oxygen or non-invasive ventilation and admission to ICU in patients with COVID-19 pneumonia.

## 2. Methods

Within 3 months (June 1^st^ to August 31^st^, 2021), during the delta surge of COVID-19, we performed a prospective, randomized, controlled, pilot study at the Department of Infectious Diseases of the University Hospital of Larissa, a large tertiary hospital, the largest public reference unit for COVID-19 in Thessaly, Greece. The population of the region of Thessaly is 732,762 (2011 national census). The study was performed according to national and international guidelines and was approved by the Ethics Committee of the hospital (13148). All patients gave informed consent. The trial was retrospectively registered in https://ClinicalTrials.gov (https://clinicaltrials.gov/ct2/show/NCT05525832).

All patients had severe COVID-19 pneumonia on admission according to the 4-category NIH clinical severity scale and were admitted in negative pressure, isolation rooms. Inclusion criteria were: [1] adult (≥18 years old) patients hospitalized primarily for COVID-19 pneumonia; and [2] a confirmed SARS-CoV-2 infection diagnosed through RT-PCR test of nasopharyngeal samples. We excluded (a) patients with confirmed SARS-CoV-2 infection who were not primarily admitted for COVID-19 pneumonia; (b) patients with use of intranasal sprays for at least two weeks prior to study enrollment; (c) sinonasal surgery within 3 months prior to study enrollment; (d) patients with sinusitis; (e) inability to perform nasopharyngeal wash; and (f) participation in other trials.

All patients were treated with the standard protocol of care for COVID-19 [6] at the Department of Infectious Diseases and were randomized into one of two groups, with the method of sequentially numbered, opaque, sealed envelopes, the normal saline group (received normal saline 0.9% solution for nasopharyngeal wash) and the control group (no treatment). In all patients, an initial baseline nasopharyngeal swab was obtained at admission and was placed in a sterile bottle of virus transport medium (10 ml tube with 3 ml medium, Biobase, Biodustry, Shandong, China) for SARS-CoV-2 nucleic acid detection (day 1). Then, an educational review of nasopharyngeal wash technique was performed. Patients were advised to fill a large syringe with the 10 ml sterile normal saline solution (NaCl 0.9%, Demo Pharmaceuticals, Krioneri, Greece), stand over a sink, keep their head straight, put the nozzle of the syringe in one nostril, try to aim the nozzle towards the back of their head, squirt the whole solution and then repeat on the other nostril. They were also advised to spit out any solution coming into their mouth and then blow their nose gently. Patients from the normal saline group were provided with eight 10 mL sterile bottles of normal saline 0.9% solution and were advised to perform nasopharyngeal wash with 10 ml of solution to each nostril, every 4 hours for a 16-hour period. Twenty-four hours after the baseline nasopharyngeal swab and 8 hours after the last nasopharyngeal wash, a second nasopharyngeal swab was collected for measurement of the viral load (day 2). This time point (24 hours) was selected to assess for an immediate effect of nasal wash while allowing for sufficient time for mucociliary clearance of the normal saline (8 hours) before the second nasopharyngeal swab was obtained. All nasopharyngeal swabs were performed by a physician who was blinded to group allocation and were collected from the same nostril for each patient.

Each sample was tested at most up to 12 hours following collection. Viral RNA was extracted from 400 *μ*L from each nasopharyngeal sample by using the commercial kit MagDEA®®Dx SV using a magLEAD®® 12gC instrument (Precision System Science Co., Matsudo City, Chiba, Japan). Detection of SARS-CoV-2 was performed by RT-PCR, by using a commercial kit that targeted the E (common for other SARS-related coronaviruses) and N (specific for SARS-CoV-2) genes (Direct SARS-CoV-2 Real-Time PCR kit, Vircell, Granada, Spain), with a threshold limit of detection of 3.5 copies per reaction for both genes. The RNase P gene region was used as an endogenous internal control for the analysis of biological samples (Direct SARS-CoV-2 Real-Time PCR kit, Vircell, Granada, Spain). A sample was considered to be SARS-CoV-2 positive, when Ct values for both the E and N genes were found to be <40, according to the recommendations of the manufacturer. Cycle threshold denoted the number of required PCR cycles before the SARS-CoV-2 viral RNA reached a detectable level. Higher N gene Ct values were indicative of lower amounts of SARS-CoV-2 viral RNA.

Patients demographic and clinical information were recorded at admission. Disease-related symptoms and potential adverse effects related to use of normal saline were also monitored. All patients were followed until hospital discharge, ICU admission, or death. Those that were discharged were re-examined 14 days after hospital discharge for RT-PCR. Manual chart review was used to gather details of the laboratory studies, course, and outcomes.

### 2.1. Statistical Analysis

All statistical analyses were performed using the Statistical Package for Social Sciences (IBM Corp. Released 2017. IBM SPSS Statistics for Macintosh, Version 25.0. Armonk, NY: IBM Corp.). Descriptive statistics were implemented in order to describe the baseline characteristics of the patients. For data with normal distribution, the means and standard deviations were reported, while, the medians and interquartile ranges were calculated for data with non-normal distributions. Paired sample *t*-tests and independent sample *t*-tests were used, as appropriate, to compare the mean Ct cycles between groups and between day 1 and 2. Fisher's exact test was used to compare the percentages in categorical data.

## 3. Results

**Figure 1:**
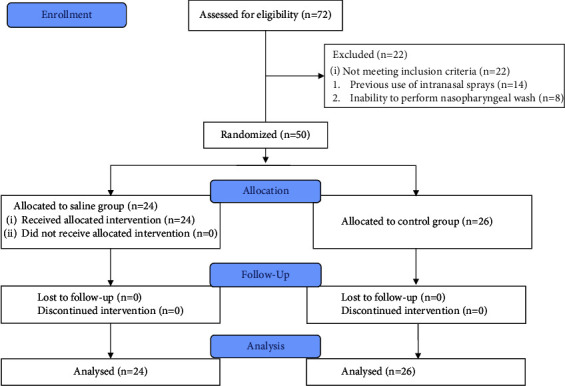
Consort flow diagram of the study.

Overall, 72 consecutive patients with COVID-19 pneumonia who were admitted in the negative pressure, isolated rooms of the Department of Infectious Diseases were screened for eligibility, and 50 were finally included in the study (24 in the saline group and 26 in the control group).  Figure 1 shows the flow of participants through the trial. The mean age was 51 ± 17 years and 28 (56%) were male. There was no difference in the duration of illness at randomization between arms. However, patients in the control arm were older (Table 1). Demographic characteristics are presented in Table 1.

### 3.1. Changes in Viral Load via Nasopharyngeal Swab Assessment

Mean baseline N target Ct values for intranasal SARS-CoV-2 were 20.9 ± 4.2 and 21.8 ± 3.5 cycles [*p*=0.4 (95%CI: −3.1 to 1.3)] in the normal saline and control group, respectively.

**Figure 2:**
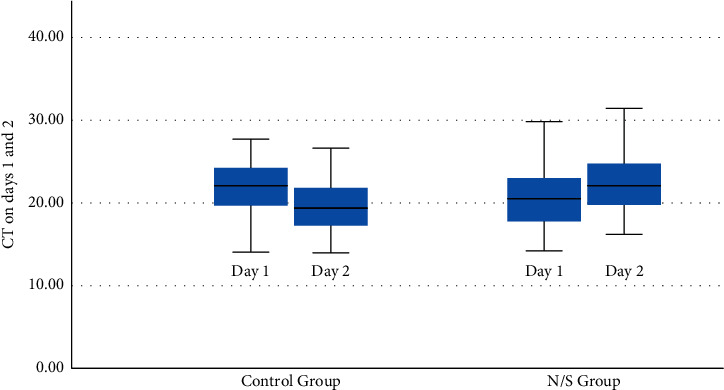
Mean N gene Ct values in day one and day two in both the groups. (CT: cycle threshold, N/S: normal saline).

In the normal saline group, mean N gene Ct values increased significantly 24 hours after the baseline measurement [ΔCt_day2−day1_ = 1.87 ± 3.11 cycles, *p*=0.007 (95 % CI: 0.55 to 3.18)], indicating a decline in SARS-CoV-2 nasopharyngeal viral load by 8.9% (Figure 2). On the other hand, a significant decrease in mean N gene Ct values was observed in the control group, indicating an increase in viral load [ΔCt_day2−day1_ = −2.12 ± 2.66, *p* < 0.001 (95%CI: −3.20 to −1.05)] by 9.7% (Figure 2). The difference in mean Ct values between the two groups 24 hours after admission and nasopharyngeal wash was 3.09 cycles (*p*=0.005, 95 % CI: 0.97 to 5.20).

Adherence was 100% in the normal saline group. There were no differences in the reported frequencies of baseline symptoms between the groups.

### 3.2. Follow Up

No statistically significant difference was observed in the need for escalation of respiratory support with high flow nasal cannula or noninvasive ventilation (2 vs. 6 in the normal saline and the control group, respectively, *p*=0.25) and admission to the ICU (0 *vs.* 3 in the normal saline and the control group, respectively, *p*=0.24). Moreover, no statistically significant difference was observed in the mean follow-up days from symptoms onset for those that were finally discharged and re-examined 14 days after hospital discharge (25.2 ± 5.4 vs 25.65 ± 5.1 for the normal saline and the control group, respectively (*p*=0.79, 95 % CI −3.02 to 3.93). At follow-up (2 weeks after hospital discharge), 15 patients in the normal saline group had a negative test versus 6 patients in the control group (*p*=0.02).

### 3.3. Adverse Events

No adverse events (nasal irritation, sensation of pain, blockage, rhinorrhea, otalgia, or nosebleeds) were observed. Two patients in the normal saline group reported minor nasal irritation. Three patients in the control group were admitted in the ICU and finally died compared to zero in the normal saline group (*p*=0.24).

## 4. Discussion

Research evidence has suggested that the indications provided by RT-PCR Ct values may afford key knowledge regarding the prognosis and consequences of SARS-CoV-2 infection thus aiding in clinical decisions [7, 8]. The Ct value is inversely related to the viral load and each 3.3 increase in the Ct value correlates with a 10-fold reduction in the starting material [7, 8]. In this pilot study, we found a significant decline in SARS-CoV-2 nasopharyngeal viral load 24 hours after the onset of nasal washes with normal saline 0.9%. On the other hand, a significant increase in viral load was observed in patients who did not perform nasopharyngeal wash. Furthermore, significantly more patients from the normal saline group had a negative test at follow-up 14 days after hospital discharge.

Previous studies in non-COVID-19 patients have demonstrated that administration of low-salt and isotonic solutions has been associated with an immediate, significant reduction in the microbial antigens and a related decline of microbial burden [9]. In contrast, hypertonic solutions were found to be only marginally capable of influencing microbial antigen concentrations [9]. Based on these data, we chose to perform nasal washes with normal saline 0.9%, which significantly decreased the nasal viral load in our patients.

The rationale for use of normal saline as inhibitor of viral replication is based on studies showing that increasing saline concentrations induce dose-dependent immediate steric hindrance in the ACE-2 receptor configuration for binding of angiotensin II, thereby inducing less virus-receptor binding [10]. Moreover, *in vitro* studies have demonstrated that NaCl depolarizes the plasma membrane of infected cells, leading to intracellular energy deprivation via affecting the ADP/ATP concentration ratio thus, preventing virus replication [11]. Furthermore, NaCl has been shown to inhibit 3Clpro, a viral enzyme regulating several steps of replication [12] and the host protease furin [13, 14]. Furin is used by the SARS-CoV-2 virus to prime its spikes towards fast fusion [13]. The proteolytic activity of furin was previously found to be inhibited by NaCl, starting at 75 mM (∼0.4%) onwards, and achieving >90% inhibition at 100 mM (0.6%), and complete inhibition at 200 mM (∼1.2%) [15]. In addition, saline shifts myeloperoxidase activity in nasal and pharyngeal epithelial or phagocytic cells to produce hypochlorous acid which exerts antiviral properties [16].

In a recent randomized open labeled trial, adults with acute upper respiratory tract infection in the last 48 h (56% from rhinovirus and 31% from coronaviruses) were randomized to perform oral gargles and nasal wash with hypertonic saline (*n* = 32) versus no intervention (*n* = 34). There was a significant reduction in the duration of illness, use of symptomatic medication, transmission of household contacts, and viral shedding measured by sequential nasal swabs (*p* < 0.05) in the hypertonic saline group [5]. In COVID-19 patients, apart from case reports [17], there is only one study by Poulas who observed a reduction of SARS-CoV-2 viral load in hospitalized patients with COVID-19 after nasopharyngeal washes with hypertonic water [18]. In contrast to our study, Poulas reported that Ct values remained stable in the negative control patients. This can be explained by the fact that nasal swabs were collected before and after a 6 h-period compared to the 24 h-period in the present study. Moreover, in the study by Poulas, the second swab was collected 30 min after three nasal washes were performed in a 6-hour period and the reduction could be purely because of the physical washing removing virus from the nasopharynx and may not reflect a true reduction in viral shedding. Our study overcomes this problem as we had an 8-hour window after the wash.

Another interesting observation was that significantly more patients from the normal saline group had a negative test at follow-up. This was an expected finding considering the significant decrease in viral load in this group, and is in accordance with previous studies with non-COVID-19 patients [5]. Moreover, the possible reduction in high flow nasal cannula use, ICU admission, and death seen in our study, suggest that nasal washes could potentially reduce serious complications of COVID-19.

We acknowledge some limitations in this study. First, it is a single-center study and should be reproduced in a multicenter one to improve general applicability. However, the aim of this pilot study was to serve as the basis for further research. Second, although all patients were middle aged, patients in the control group were older than those in the normal saline group. Third, swabs from the same person were not tested on the same RT-PCR run which may have contributed to inter-assay variability. Finally, the second RT-PCR test was performed 24 hours after hospital admission to assess for an immediate effect of nasal wash on viral load. Future studies must include a longer follow-up to assess the kinetics of viral load after nasal wash.

## 5. Conclusions

This pilot study demonstrated that nasal washes with normal saline effectively decreased the viral load during hospitalization and at follow-up. These results will be used to support the development of a larger randomized, controlled clinical trial that will definitively answer if nasal washing which is an affordable and safe strategy, can be used as an extra hygienic measure add-on to standard hygienic interventions to reduce transmission from the treated patient.

## Figures and Tables

**Table 1:** Baseline characteristics of the population studied.

Characteristics	Normal saline group	Control group	*p* value
Participants, *n*	24	26	—
Age, years (mean ± SD)	45 ± 15	56 ± 17	0.01
Male, *n* (%)	13 (54.2)	15 (57.7)	1
PaO_2_/FiO_2_, median (IQR)	325.5 (44.25)	306.5 (63.75)	0.17
Duration of symptoms prior to enrollment, days [median (IQR)]	8 (4.75)	7.5 (1.25)	0.94

*Comorbidities*			
Cardiac disease, *n*	6	10	0.37
Respiratory disease, *n*	3	5	0.7
Obesity, *n*	1	5	0.19
Change of taste or smell at enrollment, *n* (%)	7 (29.2)	5 (19.2)	0.51

SD: standard deviation, PaO_2_: partial pressure of oxygen, FiO_2_: fraction of inspired oxygen, IQR: interquartile range.

## Data Availability

The excel file with data used to support the findings of this study is available from the corresponding author upon request (pantazopoulosioannis@yahoo.com).

## References

[1] Petersen E., Ntoumi F., Hui D. S. (2021). Emergence of new SARS-CoV-2 variant of concern omicron (B.1.1.529)—highlights Africa’s research capabilities, but exposes major knowledge gaps, inequities of vaccine distribution, inadequacies in global COVID-19 response and control efforts. *International Journal of Infectious Diseases*.

[2] Zou L., Ruan F., Huang M. (2020). SARS-CoV-2 viral load in upper respiratory specimens of infected patients. *New England Journal of Medicine*.

[3] Jajou R., Mutsaers-van Oudheusden A., Verweij J. J., Rietveld A., Murk J. L. (2022). SARS-CoV-2 transmitters have more than three times higher viral loads than non-transmitters—practical use of viral load for disease control. *Journal of Clinical Virology*.

[4] Pujadas E., Chaudhry F., McBride R. (2020). SARS-CoV-2 viral load predicts COVID-19 mortality. *The Lancet Respiratory Medicine*.

[5] Ramalingam S., Graham C., Dove J., Morrice L., Sheikh A. (2019). A pilot, open labelled, randomised controlled trial of hypertonic saline nasal irrigation and gargling for the common cold. *Scientific Reports*.

[6] COVID-19 Treatment Guidelines (2021). Information on COVID-19 treatment, prevention and research [Internet]. https://www.covid19treatmentguidelines.nih.gov/.

[7] Rabaan A. A., Tirupathi R., Sule A. A. (2021). Viral dynamics and real-time RT-PCR Ct values correlation with disease severity in COVID-19. *Diagnostics*.

[8] Tom M. R., Mina M. J. (2020). To interpret the SARS-CoV-2 test, consider the cycle threshold value. *Clinical Infectious Diseases*.

[9] Woods C. M., Tan S., Ullah S., Frauenfelder C., Ooi E. H., Carney A. S. (2015). The effect of nasal irrigation formulation on the antimicrobial activity of nasal secretions. *International Forum of Allergy & Rhinology*.

[10] Guy J. L., Jackson R. M., Acharya K. R., Sturrock E. D., Hooper N. M., Turner A. J. (2003). Angiotensin-converting enzyme-2 (ACE2): comparative modeling of the active site, specificity requirements, and chloride dependence. *Biochemistry*.

[11] Machado R. R. G., Glaser T., Araujo D. B. (2021). Inhibition of severe acute respiratory syndrome coronavirus 2 replication by hypertonic saline solution in lung and kidney epithelial cells. *ACS Pharmacology and Translational Science*.

[12] Huijghebaert S., Hoste L., Vanham G. (2021). Essentials in saline pharmacology for nasal or respiratory hygiene in times of COVID-19. *European Journal of Clinical Pharmacology*.

[13] Hasan A., Paray B. A., Hussain A. (2021). A review on the cleavage priming of the spike protein on coronavirus by angiotensin-converting enzyme-2 and furin. *Journal of Biomolecular Structure and Dynamics*.

[14] Bestle D., Heindl M. R., Limburg H. (2020 Sep). TMPRSS2 and furin are both essential for proteolytic activation of SARS-CoV-2 in human airway cells. *Life Science Alliance*.

[15] Izidoro M. A., Gouvea I. E., Santos J. A. N. (2009). A study of human furin specificity using synthetic peptides derived from natural substrates, and effects of potassium ions. *Archives of Biochemistry and Biophysics*.

[16] Ramalingam S., Cai B., Wong J. (2018). Antiviral innate immune response in non-myeloid cells is augmented by chloride ions via an increase in intracellular hypochlorous acid levels. *Scientific Reports*.

[17] Rosati P., Giordano U., Concato C. (2020). Hypertonic saline nasal irrigation and gargling as an inexpensive practical adjunctive weapon to combat asymptomatic SARS-CoV-2 infections: a case report. *Trends in Medicine*.

[18] Poulas K. (2021). First report of reduced severe acute respiratory syndrome coronavirus 2 viral load after nasopharyngeal wash with hypertonic water. *Qeios*.

